# Ultrasonic imaging as a means of monitoring gonadal development in lumpfish (*Cyclopterus lumpus*)

**DOI:** 10.14814/phy2.15811

**Published:** 2023-09-21

**Authors:** Frank Thomas Mlingi, Velmurugu Puvanendran, Erik Burgerhout, Helge Tveiten, Jonna Tomkiewicz, Elin Kjørsvik, Maren Mommens

**Affiliations:** ^1^ Department of Biology Norwegian University of Science and Technology (NTNU) Trondheim Norway; ^2^ Department of Production Biology Nofima AS Tromsø Norway; ^3^ Norwegian College of Fisheries Science, UiT The Arctic University of Norway Tromsø Norway; ^4^ Institute of Aquatic Resources, Technical University of Denmark Kongens Lyngby Denmark; ^5^ Department of Breeding and Research and Development AquaGen AS Trondheim Norway; ^6^ Present address: Ode AS Stadsbygd Norway

**Keywords:** gonadal development, lumpfish, sex steroids, ultrasound

## Abstract

The commercial farming of juvenile lumpfish requires monitoring of gonadal development to achieve synchronized production. Conventional methods such as gonadosomatic index (GSI), sex hormone analyses, gonadal histology, endoscopy, and gene expression analyses are costly, invasive, and often involve sacrificing the fish. We assessed the efficiency of ultrasound as a non‐invasive method for monitoring gonadal development in lumpfish. Based on ultrasound observations, we categorized the fish into six stages; F0 to F5 for females and M0 to M5 for males, that represented maturity levels from immature to spent. Importantly, the ultrasound gonadal stages aligned with histological gonadal stages. Additionally, ultrasound stages aligned with profiles of GSI, testosterone (T), 11‐ketotestosterone, and 17β‐estradiol throughout gonadal development including the spawning period. Moreover, these parameters exhibited significant positive correlations with each other reflecting their parallel trends during gonadal development. To minimize the frequency of ultrasound usage and fish handling, we established F3 and M3/M4 as arbitrary thresholds for identifying ripe females and males, respectively. By using these thresholds, the need for regular ultrasound monitoring could be reduced during most of the rearing period. Ultrasound proves to be useful and reliable for monitoring gonadal development in lumpfish, enabling synchronized production of juvenile fish.

## 1 INTRODUCTION

In the salmon industry, sea lice are among the extremely threatening parasites, they negatively affect the fish and the environment, and the public perceptions of aquaculture, thus posing probably the greatest economic impact (Costello, 2006, 2009). Moreover, these parasites are developing resistance to chemotherapeutants further threatening the sustainability of this control method; but the use of cleaner fish to delouse salmon has emerged as an environmentally friendly alternative biological control (Aaen et al., 2015; Powell et al., 2018). Wrasses are used as cleaner fish, but due to winter dormancy and ceasing feeding at below 6°C, their delousing efficiency is limited (Powell et al., 2018). On the other hand, lumpfish demonstrate activeness through winter and spring, and this has contributed to an increased commercial production of juveniles up to 4.8 million in the UK in 2017, and more than 34 million in Norway in 2020 (Directorate of Fisheries, 2022; Powell et al., 2018). Additionally, lumpfish roe is among the non‐sturgeon caviars that are moderately priced and has a long shelf life (Farag et al., 2021).

To eliminate dependence on the wild collection of broodstock and produce lumpfish juveniles for delousing from fully domesticated broodstock, there is an increased interest towards optimizing the control of reproduction. Recent findings have shown that, environmental cues such as photoperiod and temperature can be used to control gonadal development and timing of spawning in lumpfish (Imsland et al., 2018, 2019; Pountney, Lein, et al., 2020). A successful control of reproduction will ensure closing the life cycle of lumpfish under farm conditions, leading to a sustainable breeding (Pountney, Migaud, & Davie, 2020) with a year‐round availability of juveniles for stocking in salmon cages (Powell et al., 2018). Therefore, development and validation of reliable hatchery production protocols is an integral step towards a sustainable lumpfish juvenile production (Pountney, Migaud, & Davie, 2020). Using environmental cues to control reproduction should be accompanied with monitoring of the gonadal development for more accurate prediction of ovulation (Ortenburger et al., 1996). Conventional methods for monitoring gonadal development in fish include: external secondary sexual characteristics, gonadosomatic index (GSI), sex hormones, gonadal histology, endoscopy, and gene expression (Breton & Berlinsky, 2014; Goulet et al., 1986; King & Pankhurst, 2003; Ortenburger et al., 1996; Taranger et al., 1999). The GSI, gonadal histology and gene expression analyses involve sacrifice of the non‐representative or most valuable fish, sex hormone analyses and endoscopy are less invasive but involve frequent handling which induces stress, poses health risks and compromises the fish survival, while the external secondary sexual characteristics may not be consistently apparent (Næve et al., 2018; Ortenburger et al., 1996; Swenson et al., 2007).

Ultrasound is a technique that employs interconversion of electric and acoustic energies to image internal anatomy, directly producing typically gray‐scale images for human and veterinary diagnostics (Novelo & Tiersch, 2012). As a non‐lethal, non‐invasive method, ultrasound imaging has also been successfully applied in sex determination and gonadal development monitoring in fish since the 1980s, and it's cost has continued to decrease along with an improved portability (Brizendine et al., 2018). Use of ultrasound technology for sexing and gonadal development monitoring has been reported in a range fish species for example Atlantic salmon (*Salmo salar*) (Næve et al., 2018, 2019), murray cod (*Maccullochella peelii*) (Newman et al., 2008), and striped bass (*Morone saxatilis*) (Blythe et al., 1994). Ultrasound can also be used for assessing gonadal morphological changes in monosex production, for example, ultrasound was used in rainbow trout (*Oncorhynchus mykiss*) to assess the structures of testes in sex reversed fish, and as a strategy to improve monosex production by eliminating males with testicular structural anomalies (Hliwa et al., 2014).

Recently, ultrasound was used to classify lumpfish females into: (1) immature: individuals with no visible gonads; (2) immature: small gonads, both ovarian lobes are apparent within the image; (3) maturing: significantly enlarged gonads, single ovarian lobe fills the image, at later stages of development, individual hydrating oocytes may become apparent within the ovarian tissue; (4) spawning: significantly enlarged gonads, single ovarian lobe fills the image, free hydrated oocytes apparent on the dorsal region of the ovarian lobe (Pountney, Lein, et al., 2020). However, the relationship of the ultrasound technique with the conventional methods such as GSI, histological, or sex hormone registrations has not been reported. Additionally, ultrasound monitoring of gonadal development in lumpfish males is still unavailable. Conventional methods of monitoring gonadal development are useful in determining the efficiency of ultrasound, which can even be improved to predict and estimate them. Various studies that have tested the efficiency of ultrasound technology, used conventional methods as a means of enabling better customization of the new method (Næve et al., 2018, 2019; Newman et al., 2008). Therefore, the present study was aimed at evaluating the efficiency of ultrasound technology as a non‐invasive method for monitoring gonadal development in females and males of lumpfish by incorporating GSI, gonadal histology, and plasma concentrations of sex steroids data and information as references.

## 2 MATERIALS AND METHODS

### 2.1 Ethics statement

The experiments and all fish handling protocols were approved by the Norwegian Food and Safety Authority (Mattilsynet, FOTS ID: 12164) concerning the use of animals in experiments.

### 2.2 Animals and rearing conditions

The fish used for this study were obtained from two experiments. In both experiments fish were obtained from fertilized wild broodstock eggs which were reared in a flow‐through system under continuous photoperiod (L:D = 24:0) and at about 10°C from hatching. In the first experiment (experiment 1), 18 months old, cultured lumpfish with a body weight (BW) of 697 ± 364.1 (mean ± SD) were subjected to either a short photoperiod or a short‐to‐continuous photoperiod for 6 months. In the second experiment (experiment 2), 12 months old, cultured lumpfish with a BW of 157.2 ± 32.2 g (mean ± SD) were exposed to either a natural annual photoperiod or a 9‐month compressed annual photoperiod for 16 months. In both experiments, temperature was elevated by 3°C during the final oocyte maturation phase in females to synchronize the spawning. The primary objective of these experiments was to assess the impact of photoperiod and temperature manipulations on the reproductive performance of lumpfish. The methodologies and outcomes of the experiments are summarized in a conference abstract (Mlingi et al., 2021).

### 2.3 Sampling and imaging

Sampling was conducted throughout the experiments, with more frequent sampling after temperature elevation. In experiment 1, two samplings were conducted 17 weeks apart before temperature elevation, and after temperature elevation other two samplings which were 4 weeks apart. In experiment 2, sampling was conducted approximately every 34 days before temperature elevation and every 14 days after temperature elevation. To collect the fish samples, an overdose of Tricaine methanesulfonate (165 mg L^−1^: MS‐222) was used to euthanize the fish. A fish was laid on a table laying on its right side. Each fish was scanned once per sampling, using a MyLab Alpha ultrasound unit (Esaote) with a linear array 3–13 MHz probe. The scan was performed at 5–7 MHz frequency, with focus point at 25–40 mm, and signal amplification (gain), of approximately 80%–90%. The ultrasound probe was positioned on the clavicle after lifting the pectoral fin. The probe was then moved in a posterior direction, following the left gonad. Once the gonad was clearly identified, an ultrasound image was captured along the length of the gonad and associated with an individual fish identification number for later estimation of ultrasound gonadal stage.

For females, the classification of the ovary was based on factors such as ovary size, partitioning between ovaries, and the size and hydration of oocytes. For males, the classification of the testis was determined by factors including testis size, presence of testicular lobes, shading, and testis interaction with other organs.

BW was registered using a digital weighing scale. Blood samples were collected from the caudal vessels in vacutainer tubes (BD Vacutainer LH 68 IU) and kept on ice during the sampling. The blood plasma was extracted by centrifuging the blood samples at 5000 rpm for 10 min at 4°C and stored at −20°C until radioimmunoassay (RIA) of sex steroids.

Gonads were removed from the fish, weighed (gonad weight [GW]), and used to calculate the GSI using the formula GSI (%) = (GW/total BW) × 100. The GSI was aligned with the ultrasound gonadal stages to assess the trend generated during gonadal development. The gonad tissues were cut and placed in histology cassettes (Simport Histonette Tissue Processing/Embedding Cassettes with Lid), fixed in 4% buffered formalin, and stored at 4°C until histological examination for gametogenesis.

### 2.4 Histology

Formalin fixed gonad tissues were dehydrated in a tissue processor (TP 1020‐1‐1, Leica Microsystems Nussloch GmbH) and embedded in paraffin. Sections with a thickness of 4 μm were obtained using a microtome (RM 2255, Leica Biosystems, Nussloch GmbH) and stained with hematoxylin and eosin. The stained sections were scanned at 40× magnification and examined using a digital scanner (NanoZoomer, Photonics) and the scanner software (NDP, Hamamatsu, Photonics). For each fish, 1 to 3 (depending on section size and gamete stage for ovary sections) and 10 (for testis sections) images were taken for sections from individual fish. The scanner software's track map feature was used to prevent overlapping of images taken from a single tissue.

ImageJ (version 1.53j; https://imagej.nih.gov/ij/) was utilized for observing and quantifying gamete stages. The identification of gamete stages was based on reference from our unpublished findings that described gametogenesis in females and males of lumpfish (F. T. Mlingi, V. Puvanendran, E. Burgerhout, E. Guercini, M. Mommens, Ø. J. Hansen, M. Fernández‐Míguez, P. Presa, H. Tveiten, J. Tomkiewicz, E. Kjørsvik, unpublished data). Grids of 252 crosses, each covering an area of 3.2 mm^2^ (for ovary sections) and 112 crosses (each covering an area of 0.0018 mm^2^ for testis sections), were selected and overlaid on the images using an ImageJ Grid plugin. The estimation of the number of crosses hitting gametes and the calculation of area fraction were performed to quantify the gamete stages in the histological sections. To obtain the number of crosses, an estimation method described previously (Hamilton & Megown, 2004) was used. The selected estimate was adjusted to ensure ideal counting of the crosses hitting gametes at different stages in the images.

The area fraction was defined as the proportion of the total area occupied by gametes in one specific stage relative to the total area occupied by all gamete stages in the section. A cross with its center on a tissue was considered a hit for calculating the area fraction. The total number of crosses hitting the gametes of one stage was counted and multiplied by the area of single cross. The total area of a section was determined after excluding somatic tissues and empty regions. This allowed for a more accurate calculation of the area fraction for each gamete stage. To evaluate the efficiency of ultrasound in estimating the ultrasound gonadal stage, a predominant stage was considered. This stage was identified based on having an area fraction of at least 30% of the total area fraction. This approach helped assess the correlation between the ultrasound observations and the histological gamete stages. The histology data were from experiment 2, and were aligned with the ultrasound gonadal stages to assess the ultrasound efficiency in determining the gonadal development stages.

### 2.5 Sex steroid analyses

Sex steroid analyses were conducted on the blood plasma samples. The concentrations of 17β‐estradiol (E2), testosterone (T), and 11‐ketotestosterone (11‐KT) were measured using RIA following a previously described protocol (Schulz, 1985). The assay characteristics and cross‐reactivities of E2 and T antisera (isolated from New Zealand White [NZW] rabbits) were examined by Frantzen et al. (2004). Cross‐reactivities of the 11‐KT antiserum (isolated from NZW rabbits) were examined by Johnsen et al. (2013).

In summary, to extract free (non‐conjugated) steroids, 300 μL blood plasma was mixed with 4 mL diethyl ether (DEE) in borosilicate glass tubes and subjected to vigorous shaking for 4 min. After a 2–3 min phase separation, the aqueous phase was frozen on dry ice, and the organic phase with steroids was subsequently decanted into new glass tubes and placed in a water bath at 45°C until the DEE was completely evaporated. The steroids were reconstituted by adding 900 μL of RIA‐buffer. Tritiated E2, T, and 11‐KT were from PerkinElmer (Boston, MA 02118 USA, 800‐762‐4000). During RIA, 100 μL of the extracts in duplicates were mixed with the specific tracer (50 μL) and antiserum (200 μL). After overnight incubation and subsequent precipitation, the radioactivity was measured by scintillation counting on a Tri‐Carb 2900TR (PerkinElmer Life and Analytical Sciences). For use in statistical calculations, values falling below the detection limits (0.2 ng/mL) were assigned a value equal to half of the detection limit (0.1 ng/mL). The sex steroid data were from both experiments, and were aligned with the ultrasound gonadal stages to assess the trends generated during gonadal development.

### 2.6 Statistical analyses

Statistical analyses were performed using R (version 4.0.4) with assistance of the “tidyverse” package collection, and the ggplot2 package extension GGally (Liu, 2020; West, 2019; Wickham & Grolemund, 2017). Since the GSI, T, 11‐KT and E2 data did not meet the assumptions of equal variances and normality, they were analyzed by Kruskal‐Wallis test. Multiple comparisons were conducted using the Wilcoxon Rank Sum test. Also, the correlation coefficients were calculated by the Spearman's rank correlation. The significance level was set at *p* < 0.05.

## 3 RESULTS

### 3.1 Ultrasound gonadal stages

**FIGURE 1:**
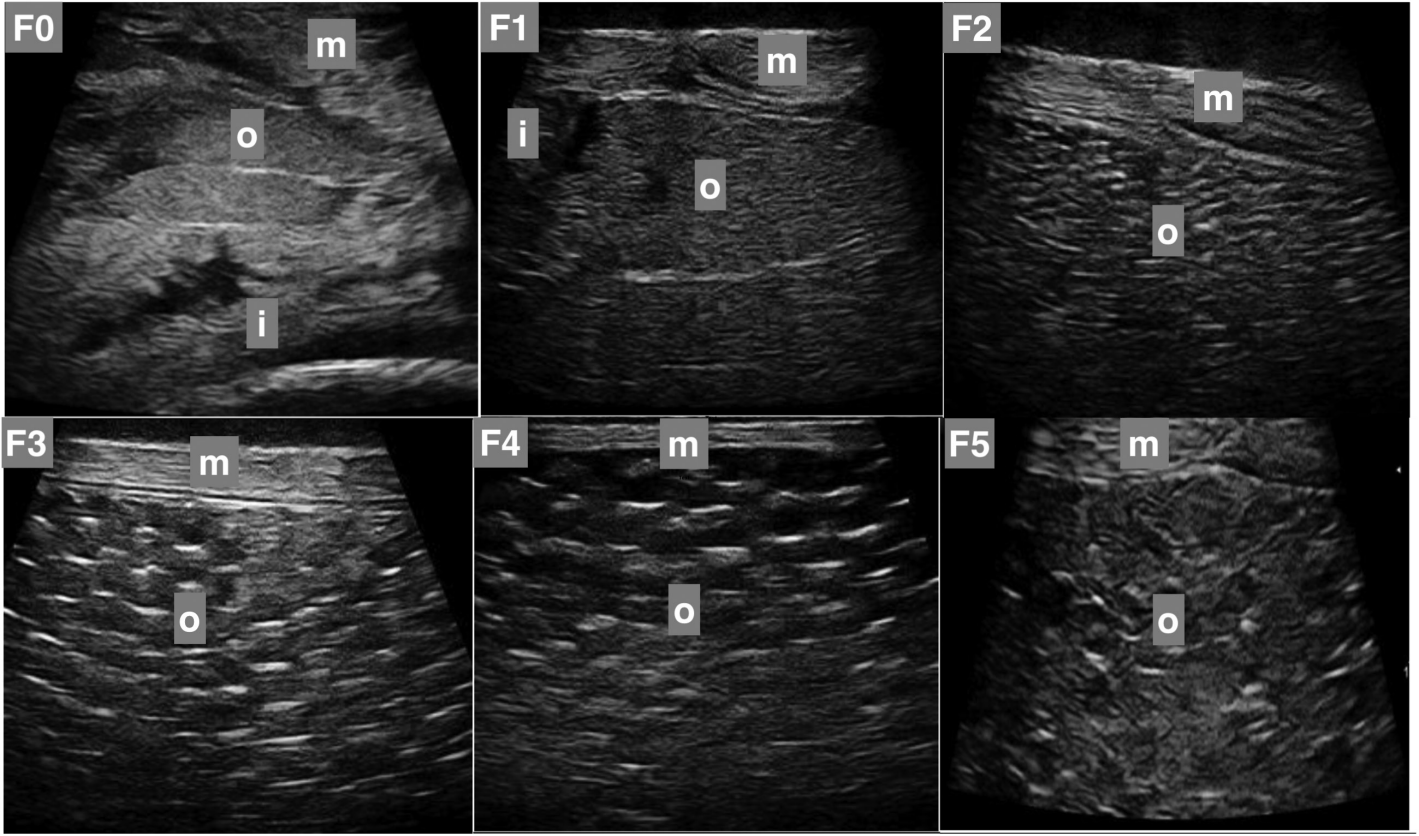
Ultrasound views of lumpfish ovaries. F0 to F5 are ultrasound gonadal stages from immature to spent ovaries. Apart from ovaries (o), intestines (i), and muscles (m) are also shown. The ultrasound gonadal stages are described in Table 1.

**FIGURE 2:**
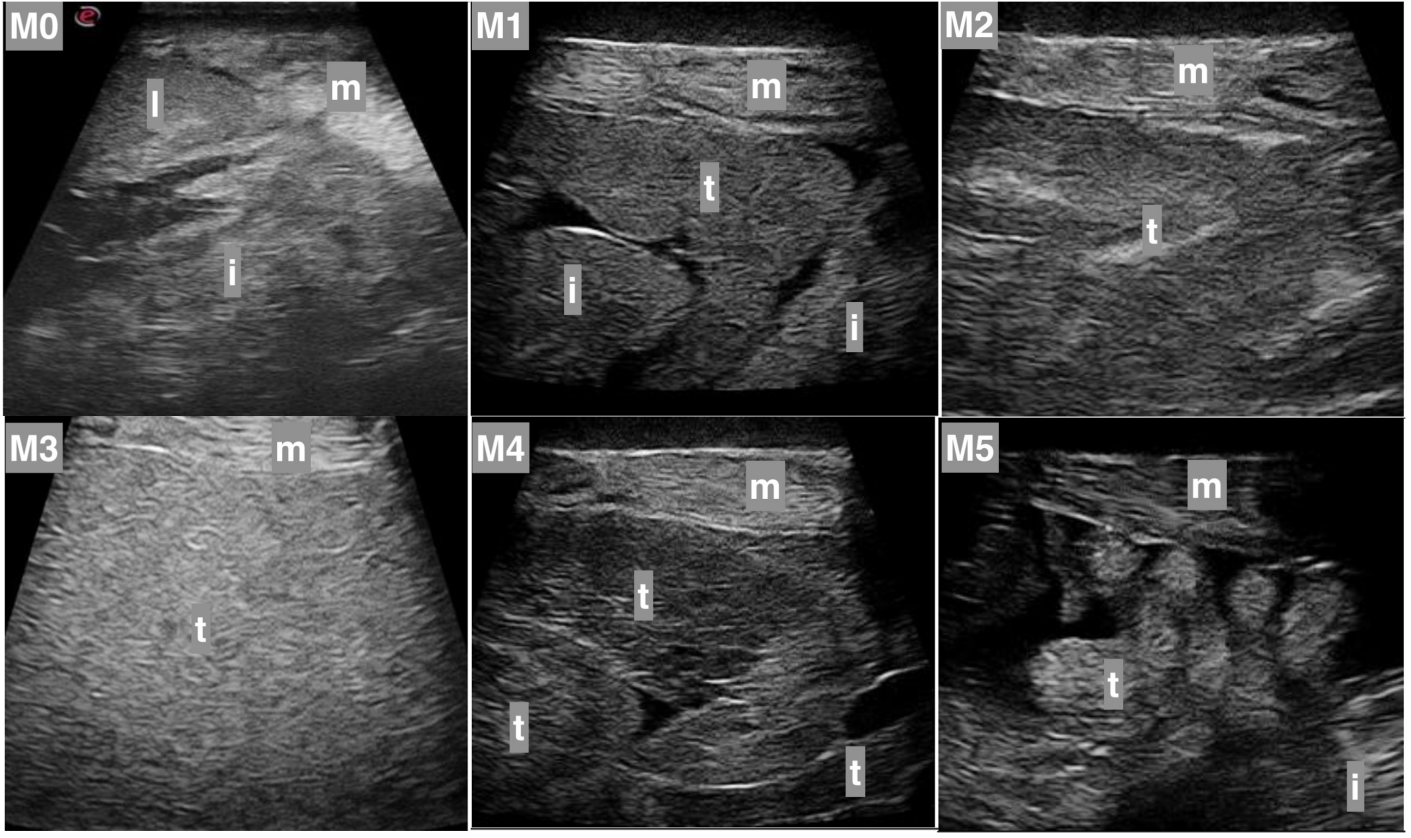
Ultrasound view of lumpfish testes. M0 to M5 are ultrasound gonadal stages from immature to spent testes. Apart from testes (t), liver (l), intestines (i), and muscles (m) are also shown. The ultrasound gonadal categories are described in Table 2.

Six ultrasound gonadal stages were established for both females (F0 to F5) and males (M0 to M5). Specific external features were observed to determine these stages, such as belly size increase, coloration of the urogenital pore, oozing of eggs in females, and body color change, running milt, and body shape change in males. The female external feature changes were visible from F2 to F4 (Figure 1; Table 1). In males, the external feature changes were visible from M3 to M5 (Figure 2; Table 2).

**TABLE 1:** Designated ultrasound gonadal stages based on the ovary and external features.

Category	Description	External feature(s)
F0 (immature)	Small ovary visible	
F1	Ovary clearly visible Visible partition between ovaries Slats in the ovaries are becoming visible	
F2	Ovaries fill more of the abdominal cavity, less visibility of other organs Slats visible Small eggs may be visible	
F3 (mature)	Large eggs clearly visible Eggs may have started to hydrate Gonads seem to have grown out	Urogenital pore has become larger and partly reddish
F4 (mature)	Hydrated eggs	Eggs easily run out through the urogenital pore
F5 (spent)	Denaturation of eggs Regeneration/slat formation	Stretch marks Urogenital pore sunken and often yellow

**TABLE 2:** Designated ultrasound gonadal stages based on the testis and external features.

Category	Description	External feature(s)
M0 (immature)	No visible testis	
M1	Visible lobe structures in testis Low degree of filling Testis first visible behind stomach Testis with and without shading	
M2	Visible lobe structure Medium degree of filling Testis visible right behind liver Testis with and without shading	
M3 (mature)	High degree of filling Testis begins right after the liver and fills much of the abdominal cavity Testis clearly shows shading	Starting to see coloration. Coloration may vary considerably in color and color strength No running milt
M4 (mature)	Lesser degree of filling	Clear coloration that varies in color and color strength Slimy and limp body Running milt
M5 (spent)	Uneven testis	Clear coloration

### 3.2 Oogenesis and spermatogenesis

This description is reported in our unpublished manuscript (F. T. Mlingi, V. Puvanendran, E. Burgerhout, E. Guercini, M. Mommens, Ø. J. Hansen, M. Fernández‐Míguez, P. Presa, H. Tveiten, J. Tomkiewicz, E. Kjørsvik, unpublished data): in females, 10 oogenic stages were identified, there were nine oocyte stages: perinucleolar; cortical alveolar; oil droplet; primary yolk; secondary yolk; tertiary yolk; maturing; matured; and ovulating oocytes, and one stage of ovulated eggs. Four ovarian development stages were established and used to categorize females based on their predominancies: primary growth (perinucleolar oocytes); secondary growth (cortical alveolar, oil droplet, primary, secondary, and tertiary yolk oocytes); oocyte maturation (maturing and matured oocytes); and ovulation (ovulating oocytes and ovulated eggs). In males, four spermatogenic cells were identified: spermatogonia, spermatocytes, spermatids, and spermatozoa, and used as the testicular development stages.

### 3.3 GSI, sex steroids and their correlations

**FIGURE 3:**
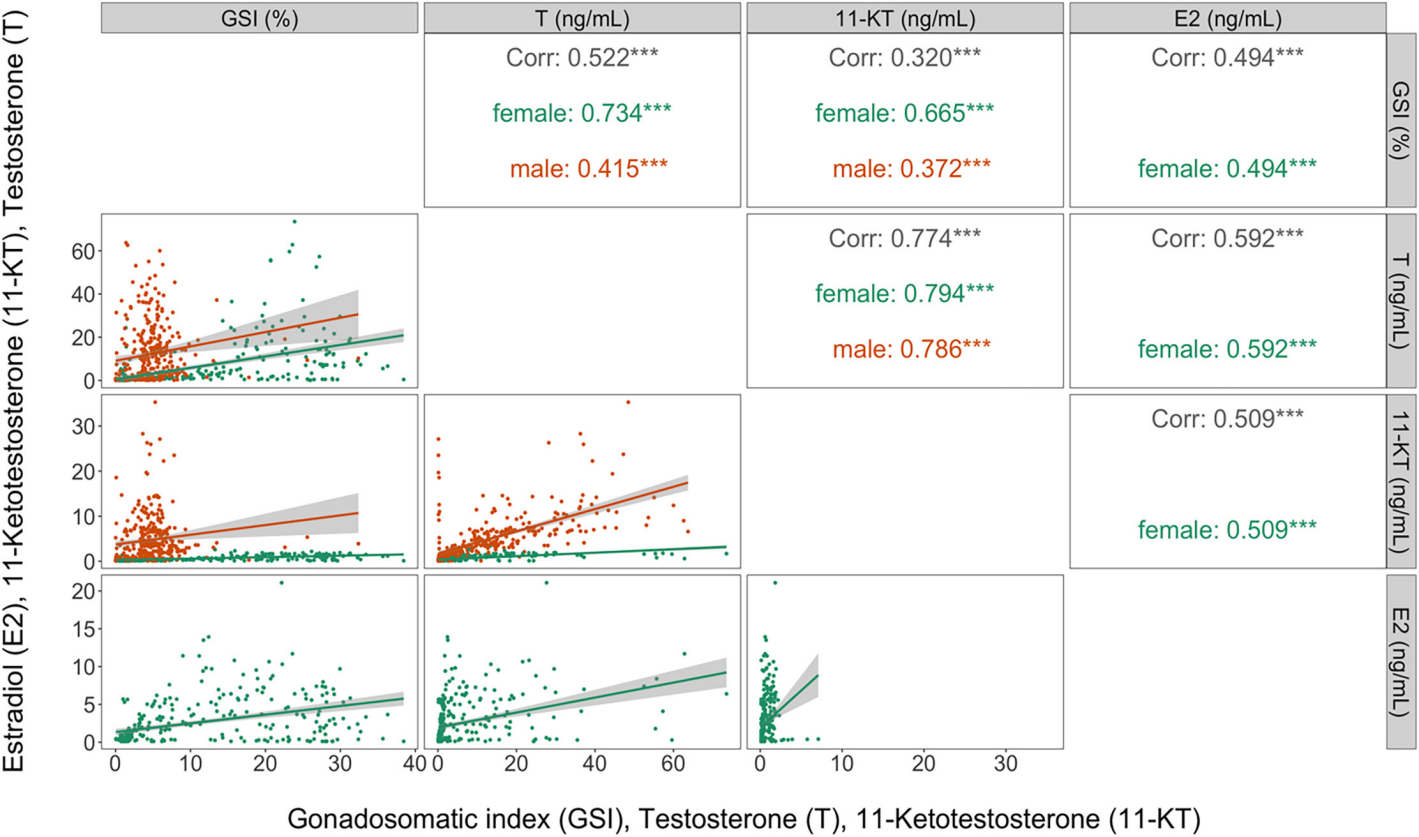
Distributions and correlations of selected sexual maturation variables, colored by sex. Scatterplots for the pairs of variables are on the lower triangle, spearman correlation coefficients are on the upper triangle of the correlation matrix (significant (*p* < 0.05) correlations are marked with asterisks). The distributions of each variable are described by density plots (females and males in one plot) on the diagonal and histograms (one plot per sex) on the left two columns. The numbers of fish per sex, and the statistical summaries for each maturation variable within each sex are shown on top of the correlation matrix by means of bars and boxplots, respectively. GSI, gonadosomatic index; T, testosterone; 11‐KT, 11‐ketotestosterone; E2, 17β‐estradiol.

Scatter plots showed that most individuals had low values in GSI, T, 11‐KT E2 (only females). GSI, T, 11‐KT, and E2 showed significant positive correlations. The correlation coefficients were generally higher in females than in males. The highest correlation coefficient was between T and 11‐KT in females (0.794), and the lowest was between 11‐KT and GSI in males (0.372). Within each sex, the highest correlation coefficients were between T and 11‐KT (0.794 and 0.786 in females and males, respectively). The lowest correlation coefficient in females was between GSI and E2 (0.494), and the lowest in males was between 11‐KT and GSI (0.372) (Figure 3).

### 3.4 Efficiency of ultrasound in estimating gonadal development stages

**FIGURE 4:**
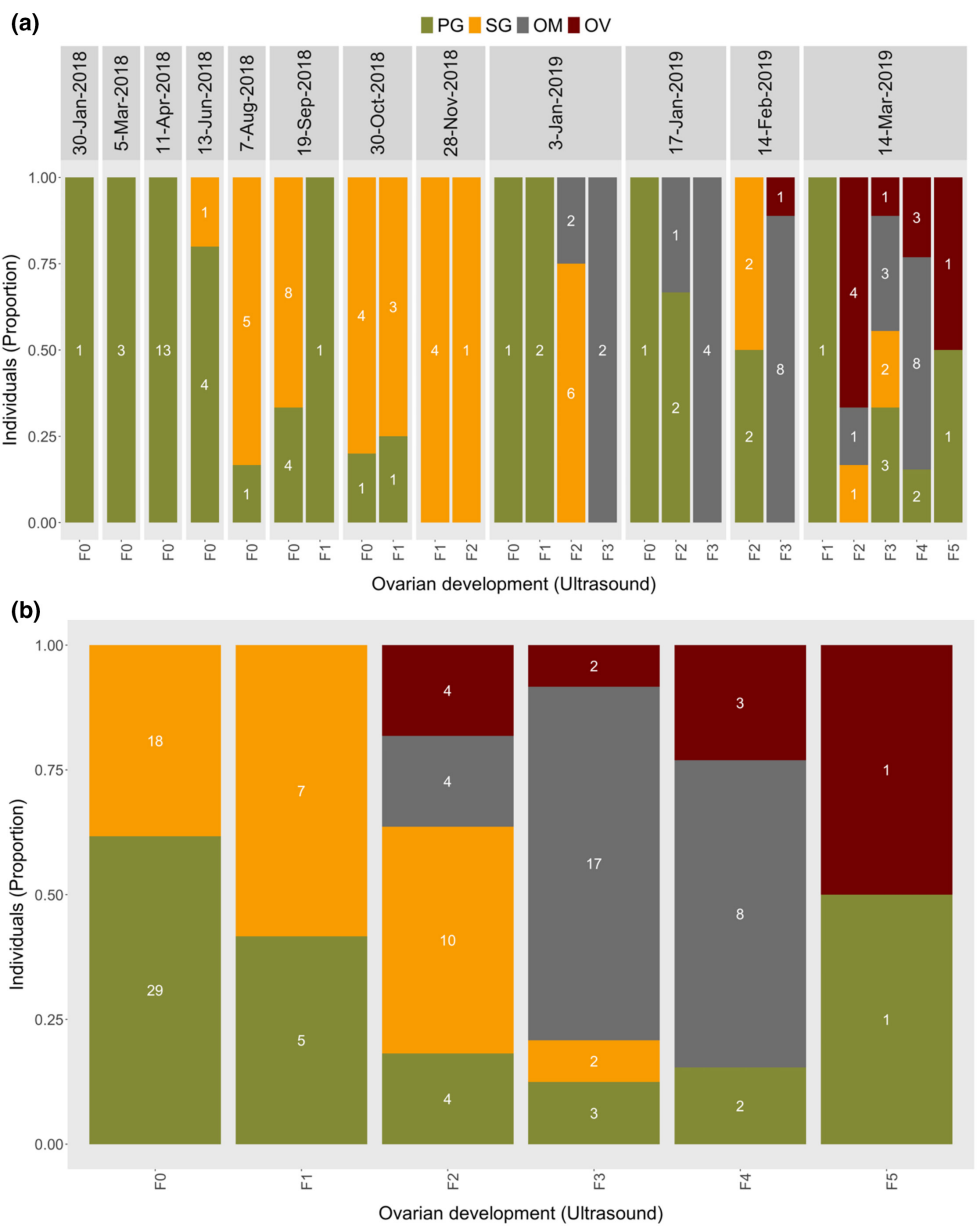
Temporal (a) and combined (b) progression of ultrasound gonadal stages in relation to predominant ovarian stages from histology. Values inside bars show the number of females in each ultrasound‐stage carrying an oogenic stage with an area fraction ≥30%. PG, primary growth; SG, secondary growth; OM, oocyte maturation; OV, ovulation.

**FIGURE 5:**
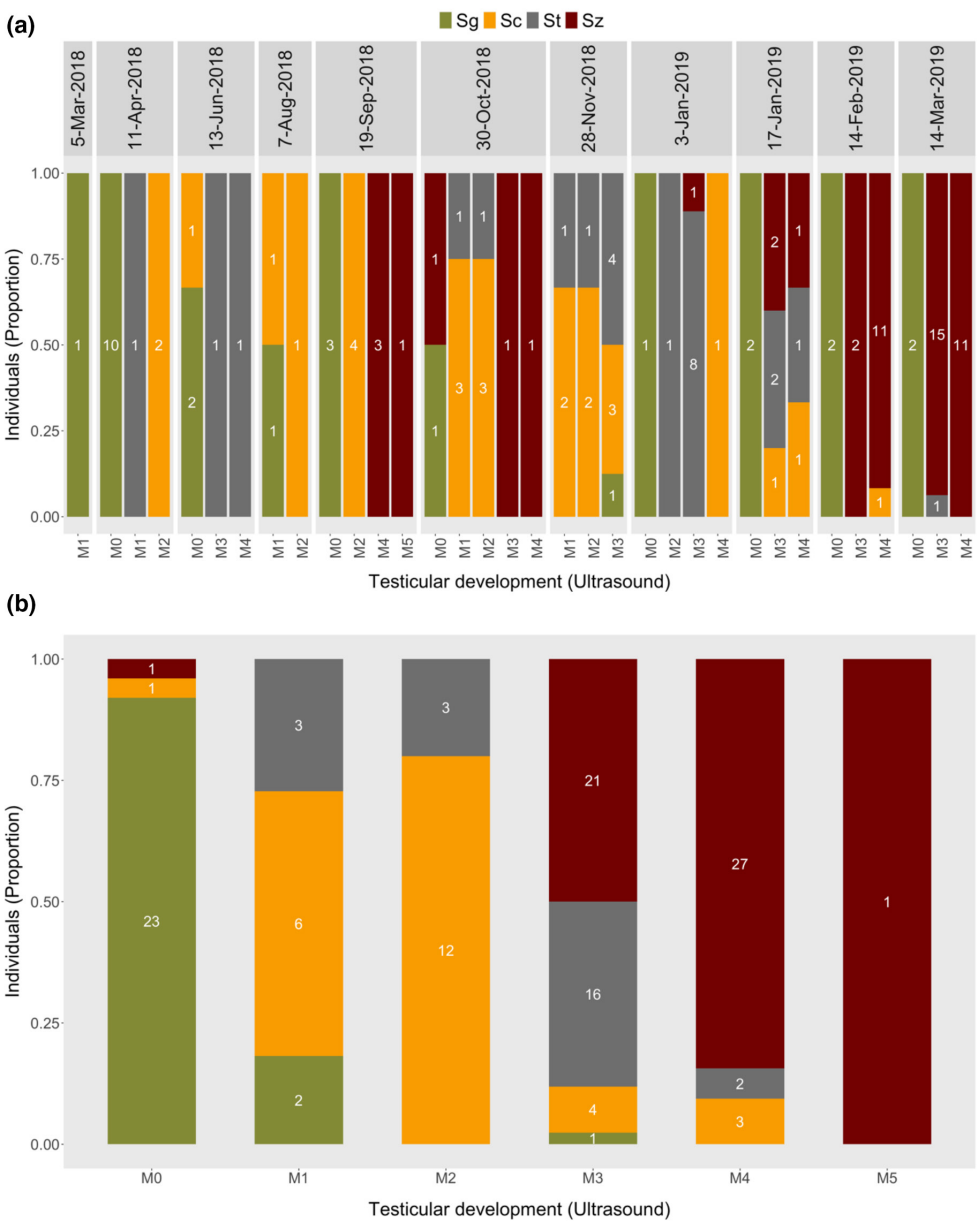
Temporal (a) and combined (b) progression of ultrasound gonadal stages in relation to predominant spermatogenic stages from testicular histology. Values inside bars show the number of males in each ultrasound stage carrying a spermatogenic stage with an area fraction ≥30%. Sg, spermatogonia; Sc, spermatocytes; St, spermatids; Sz, spermatozoa.

**FIGURE 6:**
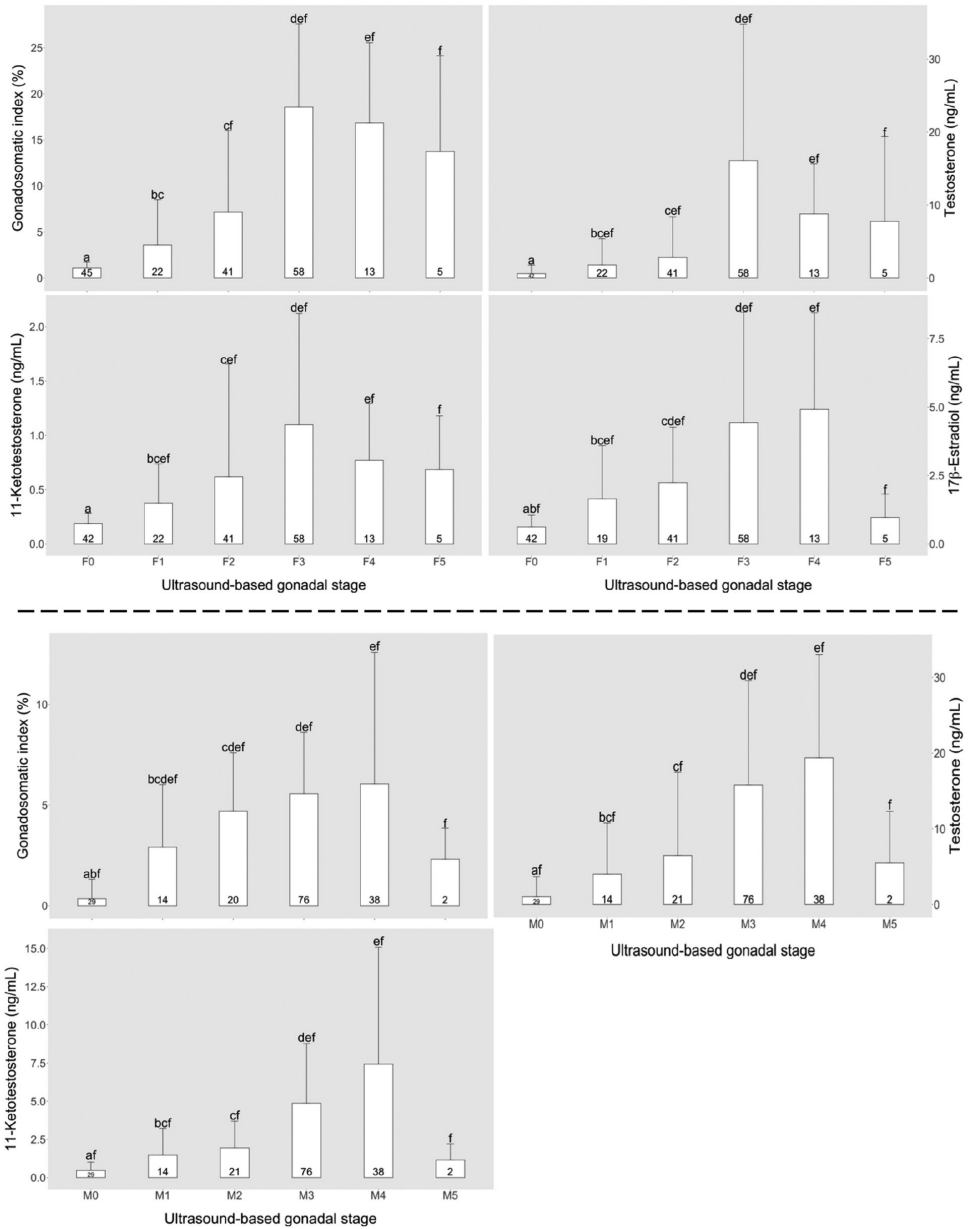
Gonadosomatic index and plasma concentrations of sex steroids at different ultrasound gonadal stages in lumpfish females (top) and males (bottom). Each ultrasound stage is assigned a unique letter (F0 & M0 = a, F1 & M1 = b, F2 & M2 = c, F3 & M3 = d, F4 & M4 = e, F5 & M5 = f), thus where an ultrasound stage carries the same letter as the next ultrasound category, it implies that difference between the two is not statistically significant. The data are presented as mean ± SD. Values inside bars represent number of fish in each ultrasound stage.

The ultrasound stages in females and males corresponded to the recruitment of more developed ovarian and testicular stages, respectively, as determined through histology. The histology sections showed that more developed oocytes were observed in higher ultrasound stages in females, while more developed spermatogenic cells were observed in higher ultrasound stages in males (Figures 4 and 5). The GSI, T, 11‐KT, and E2 concentrations also displayed patterns that increased towards specific stages in females (F3 for GSI, T and 11‐KT, and F4 for E2) and males (M4 for GSI, T, and 11‐KT) (Figure 6).

## 4 DISCUSSION

The first study that used ultrasound to estimate gonadal stages in lumpfish described four ovarian stages based on ultrasound observations only (Pountney, Lein, et al., 2020). Although Pountney, Lein, et al., 2020 provided insights into using ultrasound to determine the development stages, validation of the stages using conventional methods was not reported. We have incorporated conventional methods in our evaluation of ultrasound efficiency in estimating gonadal stages in lumpfish females and males.

The progression of ultrasound gonadal stages that followed the recruitment of more developed stages of ovary as well as the profiles of GSI, and sex steroids during ovarian development indicate that ultrasound can accurately estimate the ultrasound gonadal stage of lumpfish females. The patterns displayed by GSI, T, 11‐KT, and E2 similarly increased with the recruitment of more developed ovarian stages, but decrease in spawning females of other species (Dahle et al., 2003; Kagawa, 2013; Methven et al., 1992). In female fish, 11‐KT is implicated with oocyte growth as observed through increase in lipid accumulation, T on the other hand is the precursor of E2 which is also implicated with oocyte growth through controlling vitellogenin synthesis and secretion from the liver (Kagawa, 2013; Lubzens et al., 2010). Our findings show that the ultrasound gonadal stage “F3” can be used as an arbitrary threshold for detecting females undergoing final maturation. This will ensure a synchronized spawning because the selection of only the ready‐to‐spawn females is possible and can be supplemented with some of the external features described on Table 1. The pre‐vitellogenic oocytes in the ovary section of the female categorized as F5 (spent) could be in initial growth in preparation for the subsequent season (Milton et al., 2018). The ovary section with only ovulated eggs in the other F5 (spent) female was an indication that these ovulated unreleased eggs would likely later undergo resorption (Besseau & Faliex, 1994).

In males, 11‐KT is believed to play a role in the final stages of spermatogenesis and milt formation, also contributing to the development of secondary sexual characteristics, while T functions through feedback mechanisms on the hypothalamic and pituitary actions (Borg, 1994; Weltzien et al., 2002). The progression of ultrasound gonadal stages in males was accompanied by fluctuations in plasma concentrations of T and 11‐KT which are usually associated with the different phases of spermatogenesis. The androgens T and 11‐KT are known to increase with spermatogenesis but decline at spermiation (Schulz et al., 2010) as shown in Atlantic cod and Atlantic halibut (Dahle et al., 2003; Weltzien et al., 2002). Testicular growth as indicated by GSI also showed a known pattern that, increases during spermatogenesis, and decreases as a result of ejaculation (Hliwa et al., 2014). Therefore, increase in the blood plasma concentrations of T and 11‐KT, and the recruitment of more advanced spermatogenic stages that accompanied the progression towards more advanced ultrasound testicular stages, indicates that ultrasound was estimating the ultrasound gonadal stage of lumpfish males well. Ultrasound can thus be used to select males which are close to spawning, for example by using the arbitrary threshold “M3/M4” in this study. This will also ensure that, synchronization of spawning in males is achieved through monitoring of the testicular stages and selecting only males which are ready to spawn using the ultrasound features described on Table 2.

The positive correlations further imply that GSI, T, 11‐KT, and E2 can accurately determine the maturation status of the fish and be used to test the efficiency of ultrasound in estimating the gonadal development. Although these conventional parameters accurately determine the gonadal development of the fish, they could be of less preference compared to ultrasound due to their undesirable implications including sacrificing valuable or non‐representative individuals, and stressing the fish due to frequent handling (Næve et al., 2018; Ortenburger et al., 1996; Swenson et al., 2007). The arbitrary thresholds we suggest (F3 and M3/M4) eliminate the necessity to use ultrasound throughout the rearing period, focusing on the period close to spawning. However, care should be taken because gonadal development can vary due to differences in growth rates in fish exposed to different environments (Armstrong et al., 2004).

By discriminating immature and matured fish, a synchronized juvenile production can be achieved because spawning will be synchronized. The use of ultrasound in lumpfish broodstock management is a non‐invasive approach that will ensure optimized allocation and utilization of resources. Using this method will of course rely on the ultrasound skills and good knowledge of lumpfish anatomy by the hatchery workers, as it was similarly suggested for the Atlantic salmon and rainbow trout (Hliwa et al., 2014; Næve et al., 2019). Lastly, as argued previously for Atlantic salmon (Næve et al., 2018) ultrasound has become more accessible for field studies due to improved portability, thus it has a high potential to improve welfare in lumpfish as well, by adherence to reduction and refinement of the number of sacrificed fish and of the maturation monitoring, respectively. To improve the ultrasound estimation of maturation status in lumpfish, and the potential for use in breeding programs, the efficiency of ultrasound in estimating GW and GSI to establish an ultrasound GSI as it was for the Atlantic salmon (Næve et al., 2018, 2019) could be necessary. This is because GSI is crucial in the estimation of reproductive condition, timing and duration of spawning season, and maturity stages (Flores et al., 2019).

## 5 CONCLUSION

With the help of conventional methods, we tested the efficiency of ultrasound in estimating the ultrasound gonadal stage of female and male lumpfish. Based on our observations, we established six ultrasound gonadal stages in both females and males. To reduce the frequency of using ultrasound, we have proposed arbitrary thresholds that can be targeted for identifying ready‐to‐spawn individuals. Lastly, we proposed optimization of this non‐invasive method to accommodate estimates of GSI which is crucial in estimating the reproductive condition of an individual. Therefore, ultrasound is a non‐invasive and efficient tool that can contribute to improving synchrony in lumpfish juvenile production.

## AUTHOR CONTRIBUTIONS

**Frank Thomas Mlingi:** Investigation, data curation, formal analysis, writing‐original draft, review & editing. **Velmurugu Puvanendran:** Conceptualization, funding acquisition, methodology, resources, investigation, project administration, review & editing. **Erik Burgerhout:** Investigation, methodology, resources, review & editing. **Helge Tveiten and Elin Kjørsvik:** Conceptualization, funding acquisition, methodology, resources, investigation, supervision, review & editing. **Jonna Tomkiewicz:** Methodology, supervision, review & editing. **Maren Mommens:** Conceptualization, funding acquisition, methodology, resources, investigation, supervision, review & editing.

## FUNDING INFORMATION

This study is part of a research project called CycloBreed, whose objective is to understand the reproductive biology of lumpfish for successful selective breeding. The CycloBreed project is funded by Fiskeri‐og havbruksnæringens forskningsfinansiering (fisheries and aquaculture industry research funding) (project number 901418) https://www.fhf.no/prosjekter/prosjektbasen/901418/. The corresponding author Frank Thomas Mlingi is a Ph.D. student and was funded by the Norwegian University of Science and Technology (NTNU).

## CONFLICT OF INTEREST STATEMENT

The authors declare that there are no competing interests or personal relationships that could influence the work reported in this paper.

## Supporting information


Data S1:

Data S2:

Data S3:

## Data Availability

The data that supports the findings of this study are available in the supplementary material of this article.
